# 

**Published:** 1875-10

**Authors:** 


### Table of Contents.

### ORIGINAL COMMUNICATIONS.

Anæsthesia in Labor. *By K. P. Moore, M. D., Knoxville, Crawford county, Georgia* ........................................ 386
An Unusual Annular Contraction of the Uterus During Labor. *By E. Mason, M. D., of Wetumpka, Alabama* ........................ 389
Blood-letting. *By M. R. May, M. D., Athens, Tennessee* .............. 391
Treatment of Chills and Fever. *By D. P. Duncan, M. D., Waynesboro, Georgia* .............................................. 393
Clinic Atlanta Medical College. *By A. W. Calhoun, M. D., Professor of Diseases of Eye and Ear* ..................................... 395

### OUR EXCHANGES.

Phimosal Paraplegia ........................................ 399
Hair Restorative Testimonial—Membranous Dysmenorrhœa ........... 400
What Savages Think of Twins ................................ 401
What Important Part Does Alcohol Play in the Economy of Man ...... 402
Deformities of Spine: Posterior Angular Curvature .............. 405
The Moral of the Michigan Difficulty ......................... 410
A Simple and Effectual Plug in Nasal Hemorrhage ................ 411
A New Adhesive Plaster—No Iron in the Coloring Matter of the Blood. 412
Bilateral Section of Cervix Uteri ............................ 413
A Simple Method of Aspirating the Bladder ..................... 414
Are Hot Drinks Cooling—Uses of Southern Vanilla—The General Management of Fractures ........................................ 415
Treatment of Different Forms of Headache ...................... 418
On the Treatment of Phagedenic Gangrenous Venereal Sores ......... 423
A Question on the Physiology of the Brain—Eczema ................ 424
Complete Gangrene of the Left Kidney .......................... 425
Infantile Thumb-Sucking—Elimination of Alcohol by the Respiration .. 426
Abstracts of the More Important Papers Read Before the British Medical Association ................................................ 427
Notes of Cases of Disease of the Nervous System—Tetanus Following Menorrhagia with Purpura Hæmorrhagica ........................ 430
Blindness and Deafness Due to Tape-Worm—Insufflation in Intestinal Obstruction ................................................ 431
Biliary Calculi—Membranous Croup and its Treatment ............. 432
The Use of Baths in the Summer Complaint of Children ............ 436
Death from Musical Excitement—Rupture of Intestines with Peritonitis.. 438

### BIBLIOGRAPHICAL.

Transactions of the Medical Association of Alabama ................ 439
Transactions of the Medical and Chirurgical Faculty of Md.—Transactions of the Medical Society of N. C.—Transactions of the College of Physicians of Philadelphia—Iridotomy—A Study of the Normal Movements of the Unimpregnated Uterus ........................ 440
Anatomical Rooms—Relations of the Nervous System to Diseases of the Skin—A New Antipruritic Remedy—Fracture of Inferior Maxillary Bone—Orations and Catalogues .................................. 441

### EDITORIAL.

Editorial Correspondence from Warm Springs, N. C. .................. 441
Meeting of ex-Confederate Army and Navy Officers—Correction........ 448

